# Activation of the Nrf2/HO-1 Signaling Pathway Contributes to the Protective Effects of *Sargassum serratifolium* Extract against Oxidative Stress-Induced DNA Damage and Apoptosis in SW1353 Human Chondrocytes

**DOI:** 10.3390/ijerph15061173

**Published:** 2018-06-05

**Authors:** Cheol Park, Su Hyun Hong, Soon Shik Shin, Dae-Sung Lee, Min Ho Han, Hee-Jae Cha, Suhkmann Kim, Heui-Soo Kim, Gi-Young Kim, Eui Kyun Park, You-Jin Jeon, Yung Hyun Choi

**Affiliations:** 1 Department of Molecular Biology, College of Natural Sciences, Dongeui University, Busan 47340, Korea; parkch@deu.ac.kr; 2 Department of Biochemistry, Dongeui University College of Korean Medicine, Busan 47227, Korea; hongsh@deu.ac.kr; 3 Anti-Aging Research Center, Dongeui University, Busan 47340, Korea; 4 Department of Formula Sciences, Dongeui University College of Korean Medicine, Busan 47227, Korea; ssshin@deu.ac.kr; 5 National Marine Biodiversity Institute of Korea, Seocheon 33662, Korea; daesung@mabik.re.kr (D.-S.L.); mhhan@mabik.re.kr (M.H.H.); 6 Department of Parasitology and Genetics, Kosin University College of Medicine, Busan 49267, Korea; hcha@kosin.ac.kr; 7 Department of Chemistry, College of Natural Sciences, Center for Proteome Biophysics and Chemistry Institute for Functional Materials, Pusan National University, Busan 46241, Korea; suhkmann@pusan.ac.kr; 8 Department of Biological Sciences, College of Natural Sciences, Pusan National University, Busan 46241, Korea; khs307@pusan.ac.kr; 9 Department of Marine Life Sciences, Jeju National University, Jeju 63243, Korea; immunkim@jejunu.ac.kr (G.-Y.K.); youjinj@jejunu.ac.kr (Y.-J.J.); 10 Department of Oral Pathology and Regenerative Medicine, School of Dentistry, Institute for Hard Tissue and Biotooth Regeneration, Kyungpook National University, Daegu 41940, Korea; epark@knu.ac.kr

**Keywords:** *Sargassum serratifolium* C. Agardh, chondrocytes, oxidative stress, apoptosis, Nrf2/HO-1

## Abstract

Oxidative stress in chondrocytes plays a critical role in the pathogenesis of osteoarthritis as an important cause of articular cartilage degradation. *Sargassum serratifolium* C. Agardh, a marine brown algae, is known to have potent antioxidant activity. Nevertheless, no study has been conducted yet on the protective efficacy against oxidative stress in chondrocytes. Therefore, the aim of the current study is to investigate the mechanism of the antioxidative effect of ethanol extract of *S. serratifolium* (EESS) on DNA damage and apoptosis induced by hydrogen peroxide (H_2_O_2_) in SW1353 human chondrocytes. For this purpose, SW1353 cells exposed to H_2_O_2_ in the presence or absence of EESS were applied to cell viability assay, comet assay, immunoblotting and flow cytometry analyses. Our results showed that EESS effectively attenuated H_2_O_2_-induced cytotoxicity and DNA damage associated with the inhibition of reactive oxygen species (ROS) accumulation. EESS also weakened the mitochondria membrane permeabilization by H_2_O_2_, and recovered H_2_O_2_-induced decreased expression of anti-apoptotic Bcl-2 and pro-caspase-3, and degradation of poly (ADP-ribose) polymerase. In addition, EESS increased not only expression, but also phosphorylation of nuclear factor-erythroid 2 related factor 2 (Nrf2), and promoted the expression of heme oxygenase-1 (HO-1), a critical target enzyme of Nrf2, but decreased the expression of kelch-like ECH-associated protein-1; however, the inhibition of HO-1 activity by zinc protoporphyrin abolished the antioxidant potential induced by EESS against H_2_O_2_-mediated oxidative stress. Therefore, the results of this study suggest that the antioxidant efficacy of EESS in chondrocytes is at least involved in the Nrf2/HO-1 signaling pathway-dependent mechanism.

## 1. Introduction

When chondrocytes are damaged by a variety of oxidative stimuli, the production of inflammatory factors and the activity of proteolytic enzymes are increased, and the initiation of apoptosis with increased generation of reactive oxygen species (ROS) is promoted [1,2]. In particular, activation of proteolytic caspase cascade, as well as mitochondrial dysfunction, plays an important role in inducing the apoptosis of chondrocytes by oxidative stress [3,4]. Also, DNA damage, apoptosis, and cartilage degradation of chondrocytes due to increased ROS production may contribute to the development of osteoarthritis, as well as the senescence of chondrocytes [1,5,6]. Therefore to protect cartilage cells, the establishment of appropriate antioxidant strategies and discovery of antioxidants to protect against oxidative stress are required.

Most cells, including chondrocytes, have endogenous defense strategies to eliminate the damage caused by excessive ROS production. Among them, the nuclear transcription factor erythroid-2-like factor 2 (Nrf2)/antioxidant response element (ARE) signaling is one of the critical antioxidant systems involved in the maintenance of the redox state for the defense of intracellular oxidative stress [7,8]. One of the ARE-regulated phase II detoxifying enzymes as a rate-limiting enzyme regulated by Nrf2 is heme oxygenase-1 (HO-1), which catalyzes the degradation of heme to biliverdin, carbon oxide, and iron [9,10,11]. Above all, HO-1, the most abundant ARE in the promoter of genes regulated by Nrf2, is a major therapeutic target of Nrf2, and has been reported to be very important in preventing disease caused by oxidative stress [12,13,14].

Recently, various natural products with antioxidant activity have been attracting attention as resources for preventing oxidative stress [15]. Among them, seaweeds rich in various pharmacologically active substances may have great potential for treatment against oxidative stress. Sargassum is a genus of marine brown algae (Phaeophyceae) that is found in most seas around the world, and many coastal residents in Korea, Japan, and China have long used this as a source of food and medicine [16]. *Sargassum serratifolium* C. Agardh, a member of *Sargassum* spp., has recently been reported to have particularly potent antioxidant, anti-inflammatory, and anticancer properties [17,18,19,20]. However, studies on the protective effect of chondrocytes have not been conducted. Therefore, in this study, the antioxidant activity of ethanol extract of *S. serratifolium* (EESS) was evaluated, as part of a study to identify seaweed-derived substances that have a potent effect on chondrocyte protection against oxidative stress. To do this, we investigated the protective effect of EESS on DNA damage and apoptosis in SW1353 human chondrocytes by mimicking in vitro oxidation using pro-oxidant agent (hydrogen peroxide, H_2_O_2_) and its related mechanism, especially the expression of Nrf2/HO-1 pathway.

## 2. Materials and Methods 

### 2.1. Preparation of EESS

The EESS used in this study was provided by the National Marine Biodiversity Institute of Korea (Seocheon, Korea). For the preparation of EESS, *S. serratifolium* was collected from offshore Jeju island, (Republic of Korea) in March 2016. Collected *S. serratifolium* was washed with tap water to remove slats, epiphytes, and sand attached to the surface of the samples, and then lyophilized. The dried sample of *S. serratifolium* (170 g) was pulverized, and extracted with 70% ethanol (*v*/*v*) for 1 h (five times) by sonication. The *S. serratifolium* extract (EESS) was obtained by evaporation under vacuum. The extract was dissolved in dimethylsulfoxide (DMSO, Sigma-Aldrich Chemical Co., St. Louis, MO, USA), before use in the experiments.

### 2.2. Cell Culture and Viability Assay

SW1353 cells were obtained from the American Type Culture Collection (Manassas, VA, USA), and were cultured in Dulbecco’s modified Eagle’s medium (DMEM, WelGENE Inc., Daegu, Korea) containing 10% fetal bovine serum (FBS, WelGENE Inc.) and 100 U/mL penicillin and streptomycin (WelGENE Inc.) at 37 °C in humidified air with 5% CO_2_. The cytotoxicity of EESS to SW1353 chondrocytes was determined by 3-(4,5-dimethylthiazol-2-yl)-2,5-diphenyltetrazolium bromide (MTT) assay. In brief, SW1353 cells were treated with EESS or H_2_O_2_ (1 mM, Sigma-Aldrich Chemical Co.) alone, or pretreated with different concentrations of EESS for 1 h before H_2_O_2_ treatment. Afterward, the medium was removed, and 0.5 mg/mL of MTT (Sigma-Aldrich Chemical Co.) was added to each well, and incubated at 37 °C for 3 h. The supernatant was then replaced with an equal volume of DMSO, to dissolve blue formazan crystals for 10 min. The optical density was measured at a wavelength of 540 nm by microplate reader (Dynatech Laboratories, Chantilly, VA, USA). All experiments were performed in triplicate.

### 2.3. Detection of the Intracellular ROS Levels

The production of intracellular ROS was monitored using a cell-permeable fluorogenic probe, 5,6-carboxy-2′,7′-dichlorofluorescin diacetate (DCF-DA). Briefly, SW1353 cells were pretreated with different concentrations of EESS for 1 h and then cultured for 1 h in the presence or absence of 1 mM H_2_O_2_. The cells were harvested and stained with 10 μM DCF-DA (Sigma-Aldrich Chemical Co.) in the dark at 37 °C for 15 min. The cells were then rinsed twice with phosphate buffered saline (PBS), and 10,000 events were immediately analyzed using a flow cytometer (Becton Dickinson, San Jose, CA, USA) with an excitation wavelength of 480 nm and an emission wavelength of 525 nm. To observe the degree of ROS production by fluorescence microscopic observation, cells attached to glass coverslips were stimulated with or without H_2_O_2_ after EESS treatment. The cells were stained with 10 μM DCF-DA at 37 °C for 15 min, washed twice with PBS, and then fixed with 4% paraformaldehyde (pH 7.4) for 20 min. The fixed cells were washed twice with PBS, and analyzed by fluorescence microscopy (Carl Zeiss, Oberkochen, Germany).

### 2.4. Comet Assay

Alkaline comet analysis was performed according to a previous research method to evaluate DNA damage [21]. Following the termination of the treatment period, the cells were mixed with 0.5% low-melting-point agarose. The mixture was spread on precoated slides with normal agarose (1% in PBS) at 37 °C and cooled to solidify using ice packs for 5 min. After the solidification of the agarose, the cells were immersed in a lysis solution (2.5 M sodium chloride (NaCl), 100 mM Na-ethylenediaminetetraacetic acid (EDTA), 10 mM Tris, 1% Triton X100, and 10% DMSO (pH 10)) for 1 h at 4 °C. The slides were placed in a gel electrophoresis apparatus containing 300 mM sodium hydroxide (NaOH) and 10 mM Na-EDTA (pH 13) for 30 min to allow the DNA to unwind, and were then subjected to electrophoresis for 30 min. After electrophoresis, the slides were rinsed three times with a neutralizing buffer (0.4 M Tris, pH 7.5) for at least 5 min each, dehydrated in absolute ethanol at 4 °C, and allowed to dry. The cells were stained with 20 μg/mL of propidium iodide (PI, Sigma-Aldrich Chemical Co.). Images were then captured using a fluorescence microscope at ×200 magnification.

### 2.5. Protein Isolation and Western Blot Analysis

To extract cellular proteins, the cells were collected, washed twice with ice-cold PBS, and then lysed using a cell lysis buffer (25 mM Tris-Cl (pH 7.5), 250 mM NaCl, 5 mM Na-EDTA, 1% nonidet-P40, 1 mM phenylmethylsulfonyl fluoride, and 5 mM dithiothreitol) for 1 h before cell debris was removed by centrifugation. Protein concentration was measured using a Bio-Rad protein assay kit (Bio-Rad Laboratories, Hercules, CA, USA), and the same amounts of protein were separated by electrophoresis on sodium dodecyl sulfate (SDS)-polyacrylamide gels and transferred to polyvinylidene difluoride membranes (Schleicher and Schuell, Keene, NH, USA). The membranes were blocked with 5% non-fat dry milk for 1 h at room temperature and subsequently probed with the primary antibodies overnight with gentle agitation at 4 °C. After washing three times with Tris-buffered saline containing 0.1% Tween-20 for 5 min, the membranes were incubated with the corresponding horseradish-peroxidase-linked secondary antibodies for 2 h at room temperature. The membranes were visualized by an enhanced chemiluminescence (ECL) solution (Amersham Corp., Arlington Heights, IL, USA) and exposed to X-ray films.

### 2.6. Detection of Nuclear Morphological Changes

To observe the nuclear morphological changes, the collected cells were fixed with 3.7% paraformaldehyde in PBS for 10 min at 25 °C and air dried. After washing with PBS, the cells were stained with 1 mg/mL of 4′,6-diamidino-2-phenylindole (DAPI, Sigma-Aldrich Chemical Co.) solution (Sigma-Aldrich Chemical Co.) at room temperature for 10 min in the dark. Finally, the cells were washed twice with PBS, and the morphological changes in the nucleus were examined using a fluorescence microscope at ×400 magnification.

### 2.7. Agarose Gel Electrophoresis for DNA Fragmentation Analysis

The harvested cells were dissolved in a lysis buffer (10 mM Tris-HCl (pH 7.4), 150 mM NaCl, 5 mM EDTA, 0.5% Triton X-100, and 0.1 mg/mL proteinase K) for 30 min at room temperature. DNA from the supernatant was extracted by chloroform/phenol/isoamyl alcohol (24/25/1, *v*/*v*/*v*, Sigma-Aldrich Chemical Co.) and was precipitated by ethanol. DNA was then transferred to 1.5% agarose gel containing 0.1 µg/mL ethidium bromide (EtBr, Sigma-Aldrich Chemical Co.). Electrophoresis was then carried out at 70 V.

### 2.8. Detection of Apoptosis by Annexin V Staining

For the quantitative evaluation of apoptosis, the annexin V-fluorescein isothiocyanate (FITC) and PI dual staining technique were employed. Briefly, the cells were collected and the suspension was made in the binding buffer (Becton Dickinson, San Jose, CA, USA). Subsequently, the cells were stained using an Annexin V-FITC Apoptosis Detection Kit (Becton Dickinson) for 20 min in the dark according to the manufacturer’s instructions. After the final incubation, at least 10,000 cells were analyzed from each sample using a flow cytometer, and the degree of apoptosis was quantified as a percentage of the annexin V-positive and PI-negative (annexin V^+^/PI^−^) cells.

### 2.9. Measurement of the Mitochondrial Membrane Potential (MMP)

Following the termination of treatment, the changes in the MMP (Δψm) were assessed using fluorescent, lipophilic, and cationic probes, as well as 5,5′,6,6′-tetrachloro-1,1′,3,3′-tetraethyl-imidacarbocyanine iodide (JC-1, Sigma-Aldrich Chemical Co.), as recommended by the manufacturer’s guidelines. The cells were then collected and rinsed with cold PBS and then stained with 10 μM JC-1 for 30 min at 37 °C in the dark. After washing with PBS to remove the unbound dye, the green fluorescence intensities from the JC1 monomer (with a 488 nm excitation) and the red fluorescence intensities from the aggregated form of JC1 (with a 575 nm emission) in the cells were measured using a flow cytometer (Becton Dickinson), following the manufacturer’s protocol.

### 2.10. Statistical Analysis

All experimental results are presented as mean ± SD. The statistical significance of the data was tested using the Graphpad Prism software (one-way analysis of variance (ANOVA) and Tukey’s post hoc test). *p* values < 0.05 were considered a significant difference.

## 3. Results

### 3.1. EESS Inhibits H_2_O_2_-Induced Cytotoxicity in SW1353 Chondrocytes

**Figure 1:**
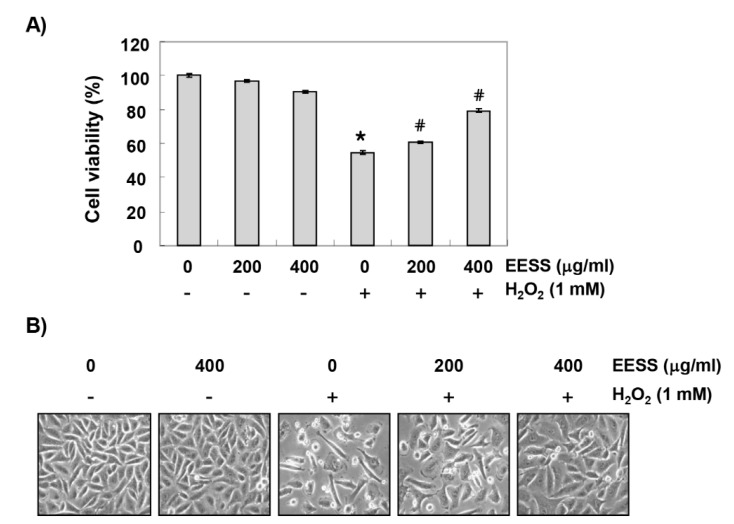
Effects of EESS (ethanol extract of *S. serratifolium*) on the H_2_O_2_-induced cytotoxicity in SW1353 chondrocytes. Cells were pretreated with the indicated concentrations of EESS for 1 h, and then incubated with or without 1 mM H_2_O_2_ for 24 h. (**A**) Cell viability was assessed by MTT (3-(4,5-dimethylthiazol-2-yl)-2,5-diphenyltetrazolium bromide) assay. The results are the means ± SD obtained from three independent experiments (* *p* < 0.05 compared with the control group; ^#^ *p* < 0.05 compared with the H_2_O_2_-treated group); (**B**) Cell morphological changes were monitored by an inverted microscope. Representative photomicrographs of the morphological changes are presented (magnification, ×200).

In order to establish the experimental conditions, SW1353 cells were treated with various concentrations of EESS, and cell viability was examined by MTT assay. The cytotoxic effect of EESS was not induced at concentrations up to 400 µg/mL, but cell viability was gradually decreased at concentrations above that (data not shown). Therefore, the maximum concentration of EESS of 400 µg/mL was chosen to study the cytoprotective effect of EESS on cell damage by H_2_O_2_. To test the protective effect of EESS on H_2_O_2_-induced cytotoxicity, SW1353 cells were treated with 200 and 400 µg/mL EESS for 1 h before 1 mM H_2_O_2_ treatment, and cultured for 24 h. Figure 1A shows that pretreatment with EESS significantly prevented the loss of cell viability in H_2_O_2_-treated SW1353 cells in a concentration-dependent manner. In addition, the morphological changes of the cells following H_2_O_2_ treatment were also restored to the control level in the pretreated cells of EESS (Figure 1B), indicating that EESS protects against cytotoxicity caused by oxidative stress.

### 3.2. EESS Suppresses H_2_O_2_-Induced Generation of ROS and DNA Damage in SW1353 Chondrocytes 

**Figure 2:**
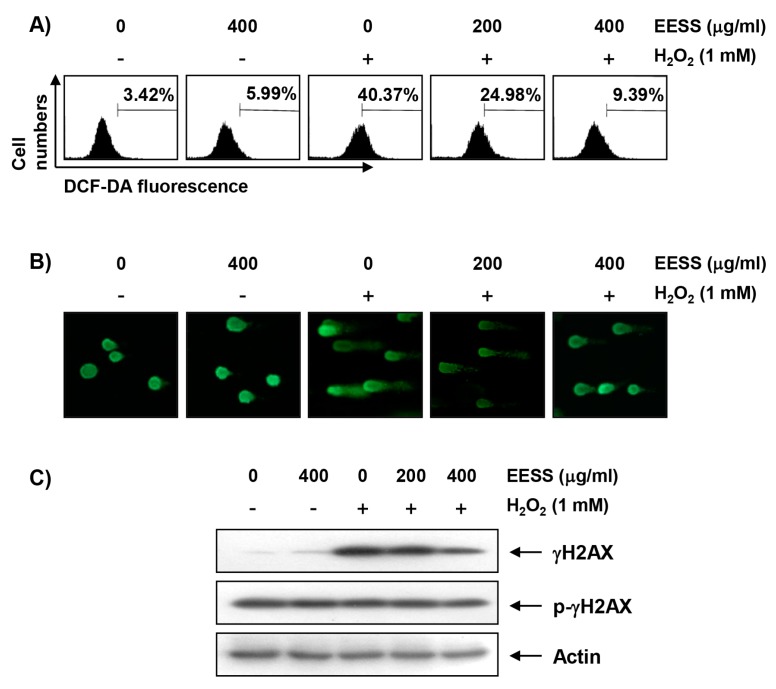
Protection of H_2_O_2_-induced ROS generation and DNA damage by EESS in SW1353 chondrocytes. (**A**) Cells were pretreated with the indicated concentrations of EESS for 1 h, and then stimulated with or without 1 mM H_2_O_2_ for 1 h. The cells were incubated at 37 °C in the dark for 20 min with culture medium containing 10 µM DCF-DA to monitor ROS production. The degree of ROS production was measured by flow cytometer. The data are the means of the two different experiments; (**B**,**C**) Cells were pretreated with EESS for 1 h, and cultured in the presence or absence of H_2_O_2_ for 24 h. (**B**) To detect cellular DNA damage, the comet assay was performed, and representative photographs of the comets were taken by fluorescence microscopy (original magnification, 200×); (**C**) The cells were lysed, and then equal amounts of cell lysates were separated on SDS-polyacrylamide gels, and transferred to membranes. The membranes were probed with specific antibodies against γH2AX and p-γH2AX, and the proteins were visualized using an ECL detection system. Actin was used as an internal control.

The antioxidant effect of EESS in SW1353 cells was determined by measuring the level of intracellular ROS reduction using fluorescent probe DCF-DA. Our flow cytometry results indicated that the level of ROS gradually increased with the treatment of H_2_O_2_, peaked at 1 h, and decreased thereafter (data not shown). However, the increase in ROS content in SW1353 cells treated with H_2_O_2_ was dramatically reduced by the addition of EESS (Figure 2A). A comet assay, which is commonly used to assess DNA strand breaks [21], was performed to assess whether the inhibitory effects of EESS on H_2_O_2_-induced viability reduction and ROS production are associated with DNA damage protection. Figure 2B shows that no smeared pattern of nuclear DNA was observed in cells treated with EESS alone as control cells. However, in H_2_O_2_-treated cells, an obvious DNA tail was observed, and these phenomena were reduced in the EESS pretreatment condition. Consistent with the results of the comet assay, the phosphorylation of γH2AX at serine 139, a biomarker of DNA double strand breaks [22], was greatly increased in H_2_O_2_-treated cells, but the degree of phosphorylation was gradually decreased with increasing EESS pretreatment concentration (Figure 2C).

### 3.3. EESS Attenuates H_2_O_2_-Induced Mitochondrial Dysfunction and Apoptosis in SW1353 Chondrocytes

**Figure 3:**
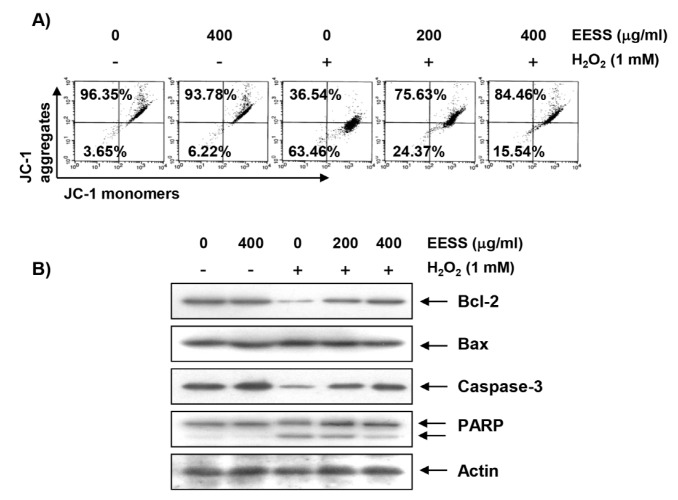
Attenuation of H_2_O_2_-induced mitochondrial dysfunction and changes of apoptosis-related proteins by EESS in SW1353 chondrocytes. Cells were pretreated with the indicated concentrations of EESS for 1 h, and then stimulated with or without 1 mM H_2_O_2_ for 24 h. (**A**) The cells were collected and incubated with 10 µM JC-1 for 20 min at 37 °C in the dark. The cells were then washed once with PBS, and the values of MMP were evaluated by flow cytometry. The data are the means of the two different experiments; (**B**) The cellular proteins were separated by SDS-polyacrylamide gel electrophoresis, and then transferred to membranes. The membranes were probed with the indicated antibodies. Proteins were visualized using an ECL detection system. Actin was used as an internal control.

**Figure 4:**
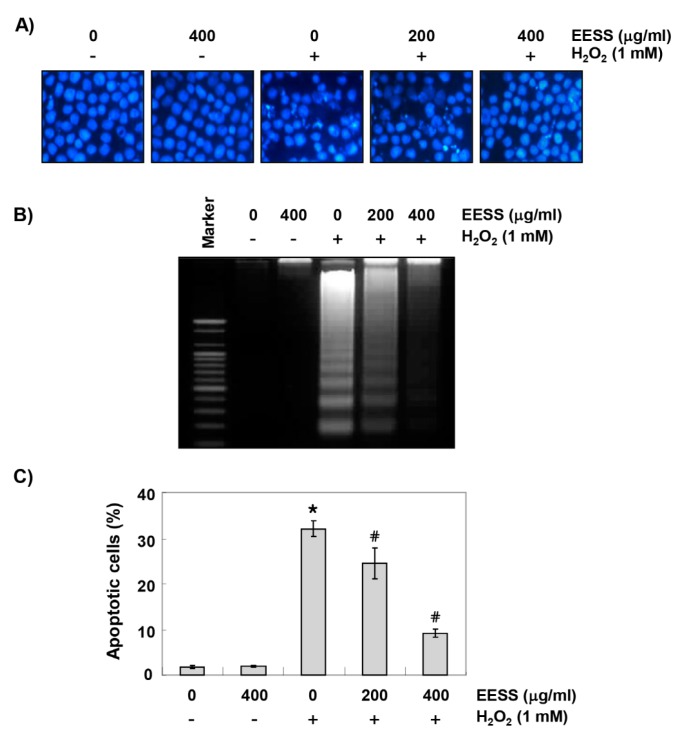
Suppression of H_2_O_2_-induced apoptosis by EESS in SW1353 chondrocytes. Cells were pretreated with or without EESS for 1 h, prior to 1 mM µM H_2_O_2_ for 24 h. (**A**) The cells were fixed and stained with DAPI solution. After 10 min incubation at room temperature, the stained nuclei were observed using a fluorescence microscope (original magnification, 400×); (**B**) DNA fragmentation was analyzed by extracting genomic DNA, electrophoresis in a 1.5% agarose gel, and then visualized by EtBr staining. (**C**) The cells were collected and stained with annexin-V and PI, and the percentages of apoptotic cells were then analyzed using flow cytometric analysis. The results are the means ± SD obtained from three independent experiments (* *p* < 0.05 compared with the control group; ^#^ *p* < 0.05 compared with the H_2_O_2_-treated group).

We next compared the values of MMP by JC1 staining to investigate the effect of EESS on mitochondrial dysfunction in H_2_O_2_-treated SW1353 cells. According to the results of flow cytometry analysis, the loss of MMP was greatly increased in H_2_O_2_-treated cells, but in the cells pretreated with EESS, the degree of such loss was concentration-dependently reduced by EESS treatment (Figure 3A). Although the expression of pro-apoptotic Bax protein was not significantly altered by H_2_O_2_ treatment, the expression of anti-apoptotic Bcl-2 and pro-caspase-3 was significantly decreased, and the degradation of poly (ADP-ribose) polymerase (PARP) was also increased. However, the reduced expression of Bcl-2 and pro-caspase-3, and increased degradation of PARP by H_2_O_2_ were relatively preserved in EESS-pretreated cells (Figure 3B). Furthermore, chromatin condensation and DNA fragmentation, which are observed in cells with typical apoptosis, were clearly observed in H_2_O_2_-treated cells; but these phenomena were markedly attenuated by the pretreatment of EESS (Figure 4A,B). As well as supporting these results, we confirmed that EESS significantly inhibited the induction of apoptosis by H_2_O_2_ through flow cytometry analysis (Figure 4C).

### 3.4. EESS Induces the Expression of Nrf2 and HO-1 in SW1353 Chondrocytes

**Figure 5:**
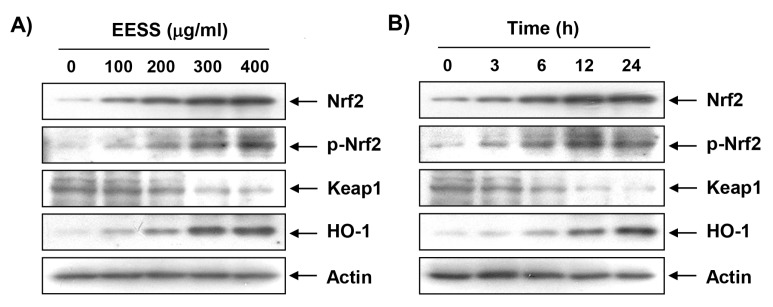
Induction of Nrf2 and HO-1 by EESS in SW1353 chondrocytes. Cells were treated with (**A**) the indicated concentrations of EESS for 24 h; or (**B**) 400 µg/mL EESS for the indicated times. The cells were lysed, and cellular proteins were separated by SDS-polyacrylamide gel electrophoresis, and transferred to membranes. The membranes were probed with the indicated antibodies. Proteins were visualized using an ECL detection system. Actin was used as an internal control.

To investigate whether antioxidant activity of EESS in SW1353 cells correlates with the activation of Nrf2/ARE signaling, the effect of EESS on the expression of Nrf2 and its regulated gene HO-1 was examined. Figure 5 shows that increasing the treatment time and concentration of EESS increased the expression of Nrf2 and HO-1 protein, but conversely, EESS treatment decreased kelch-like ECH-associated protein-1 (Keap1) expression. In particular, phosphorylation at serine 40, which is important for the activation and stabilization of Nrf2, increased with EESS treatment, demonstrating that EESS activates Nrf2/HO-1 signaling in SW1353 cells.

### 3.5. The Protective Effect of EESS on H_2_O_2_ Treatment in SW1353 Chondrocytes Involves Activation of Nrf2/HO-1 Signaling

**Figure 6:**
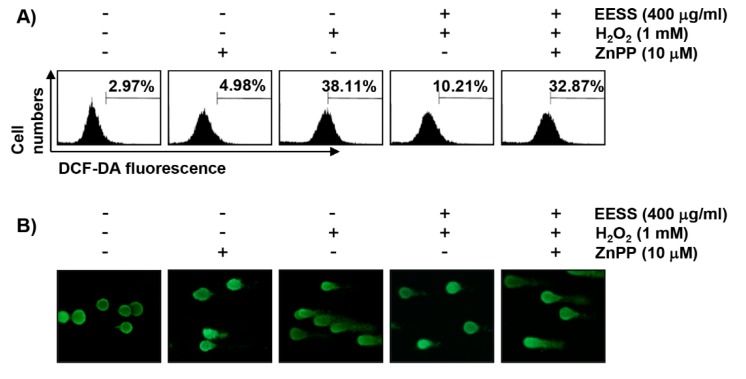

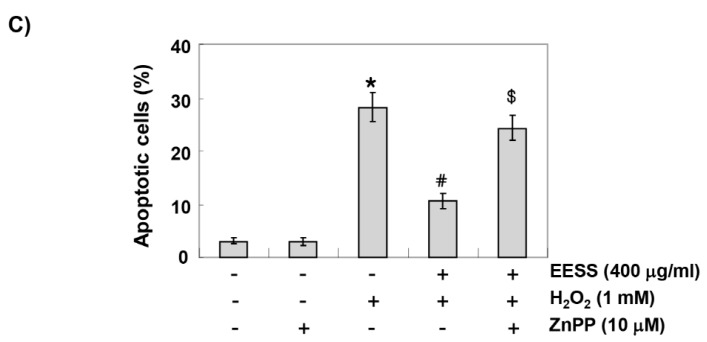
Effects of EESS on H_2_O_2_-induced ROS generation, DNA damage, and apoptosis in SW1353 chondrocytes. The cells were pretreated for 1 h with 400 µg/mL EESS, and then treated for 24 h with or without 1 mM H_2_O_2_ in the absence or presence of 10 µM ZnPP. (**A**) After incubation with 10 µM JC-1 for 20 min, JC-1 fluorescence intensity was detected by flow cytometry. Each point represents the mean of two independent experiments; (**B**) The comet assay was performed to detect cellular DNA damage, and representative photographs of the comets were taken by fluorescence microscopy (original magnification, 200×); (**C**) To quantify the degree of apoptosis, the cells were stained with FITC-conjugated Annexin-V and PI for flow cytometry analysis. The data are represented as the means ± SD obtained from three independent experiments (* *p* < 0.05 compared with the control group; ^#^ *p* < 0.05 compared with the H_2_O_2_-treated group; ^$^ *p* < 0.05 compared with H_2_O_2_ and the EESS-treated group).

In order to examine the role of Nrf2/HO-1 signaling in EESS-mediated protective effects against oxidative stress in SW1353 cells, zinc protoporphyrin (ZnPP), a specific inhibitor of HO-1, was used to inhibit HO-1 activity. Our flow cytometry results showed that in the presence of ZnPP, the protective effect of H_2_O_2_-induced MMP loss by EESS was significantly diminished (Figure 6A). In addition, the comet assay demonstrated that in the presence of ZnPP, the protective effect of H_2_O_2_-induced DNA damage of EESS was attenuated (Figure 6B). Together with these results, we also found that ZnPP reversed the EESS-mediated inhibition of H_2_O_2_-induced apoptotic activity in SW1353 cells (Figure 6C), suggesting that activation of Nrf2/HO-1 signaling is involved in the protective activity of EESS against oxidative stress.

## 4. Discussion

In the current study, we investigated the protective effect of ethanol extract of *S. serratifolium* (EESS), a marine brown algae, on H_2_O_2_-induced oxidative stress in SW1353 chondrocytes. Our results demonstrated that EESS showed potent protective activity, indicating increased cell viability, and reduced DNA damage and apoptosis in H_2_O_2_-treated SW1353 cells, which was associated with the inhibition of ROS production. In addition, EESS enhanced the activation of the Nrf2/HO-1 signaling pathway and the inhibition of HO-1 activity abolished the protective effect of EESS, indicating that the beneficial effect of EESS on SW1353 cells is mediated by activation of at least Nrf2/HO-1 antioxidant signaling. Although the antioxidant efficacy of *S. serratifolium* has been reported in several articles, this is the first study to show that the Nrf2/HO-1 pathway contributes to the protective effect of oxidative stress by *S. serratifolium* on chondrocytes.

Oxidative stress is considered to be a major cause of the dysfunction of chondrocytes, and many studies have shown that the use of natural products with antioxidant properties is adequate to prevent the functional damage of chondrocytes by oxidative stress [1,8,23]. Mitochondria are the major intracellular organelles responsible for the generation of ROS, and are also a major target of ROS [24,25,26]. Excessive ROS production, along with mitochondrial dysfunction, can lead to DNA damage, and also acts as a signaling molecule to promote apoptosis [27,28]. Therefore, inhibition or scavenging of ROS can protect against the pathological causes of various diseases associated with chondrocyte dysfunction caused by oxidative stress. In this study, we confirmed by MTT assay that EESS significantly protected SW1353 cells from oxidative stress. Using comet and immunoloting assays, we also demonstrated that EESS could block DNA damage due to oxidative stress through the inhibition of DNA tail formation and γH2AX phosphorylation, which were associated with decreased ROS production. Although further experiments on the correct production pathway of ROS are required, these results suggest that the inhibition of excessive ROS production by EESS contributes to the attenuation of H_2_O_2_-induced proliferation inhibition and DNA damage in SW1353 chondrocytes.

In general, apoptosis inducing pathway can be divided into mitochondria-dependent intrinsic and death receptor-mediated extrinsic apoptotic signaling pathways. Apoptosis due to excessive ROS production in chondrocytes is a mitochondrial-dependent pathway leading to the loss of MMP, the first event that begins through mitochondria membrane permeabilization, which is recognized as an indicator of mitochondrial damage [24,28,29]. Reduced MMP induces the release of death-promoting factors such as cytochrome *c* from the mitochondria to the cytoplasm, and cytochrome *c* in the cytoplasm forms apoptosomes by binding to apoptotic protein activating factor 1 [30,31]. Apoptosomes sequentially activate caspases-9, a potent stimulant of intrinsic apoptosis pathway. Activated caspase-9 accelerates activation of effector caspases, such as caspase-3 and -7, to destroy the various substrate proteins necessary for cell survival, and eventually induce apoptosis. Additionally, pro-apoptotic proteins, such as Bax, belonging to the Bcl-2 family members can translocate to the mitochondria, destroy the MMP, and open mitochondrial pores to release induce cytochrome *c*, while anti-apoptotic proteins such as Bcl-2 reverse this process [32,33]. The results of this study show that EESS suppressed H_2_O_2_-induced loss of MMP in SW1353 cells, and EESS co-treatment also effectively reversed the increase in Bax/Bcl-2 ratio after H_2_O_2_ treatment. With these results, pretreatment with EESS restored the decreased expression of pro-caspase-3 by H_2_O_2_ treatment to the control level, and inhibited the degradation of PARP, a substrate protein of activated caspase-3. In addition, we confirmed that H_2_O_2_-induced apoptosis was suppressed by EESS through nuclear morphological changes, DNA fragmentation, and flow cytometry analysis. These results imply that EESS was able to inhibit apoptosis through preservation of mitochondrial function, while eliminating ROS produced by oxidative stress in SW1353 chondrocytes.

Accumulated evidence indicates that Nrf2 plays an important role in protecting against oxidative damage by promoting the expression of antioxidant enzymes in most cells, including chondrocytes [7,8,34]. For example, it has recently been reported that the activation of Nrf2 by several natural products protected apoptosis and cartilage damage by suppressing the production of catabolic and inflammatory mediators by chondrocytes [9,11]. Moreover, HO-1, one of the target genes involved in the activation of Nrf2 signaling, has been shown to reduce the onset of osteoarthritis [8,10]. Collectively, these results suggest that Nrf2 may be an attractive target for chondrocyte protection by oxidative stress. Under physiological conditions, Nrf2 binds to Keap1 and is sequestered in the cytoplasm, but when subjected to a situation that responds to oxidative stress, Nrf2 is disassociated from Keap1, and then translocates to the nucleus to activate the transcription of the cytoprotective genes, including HO-1 [11,12]. In this process, phosphorylation of Nrf2 is accompanied by upstream kinases, and phosphorylation of Nrf2 is an essential process for the transcriptional activation of its target genes [8,14]. The results of this study show that the expression, as well as the phosphorylation, of Nrf2 was gradually increased in SW1353 chondrocytes treated with EESS, but in contrast, the expression of Keap1, a main negative regulator of NRF2, was decreased, indicating that EESS activates Nrf2/HO-1 signaling in SW1353 cells. Therefore, we investigated whether the activation of the Nrf2/HO-1 antioxidant pathway is involved in the protective effect of EESS on oxidative stress in SW1353 chondrocytes, and found that artificially blocking HO-1 activity by ZnPP, a representative HO-1 inhibitor, markedly weakened the protective effect of EESS on H_2_O_2_-induced ROS production. In addition, the ability of EESS to protect DNA damage and apoptosis by H_2_O_2_ disappeared in the presence of ZnPP, collectively, supporting that the Nrf2/HO-1 signaling pathway may contribute to the protective effect of EESS on oxidative stress in SW1353 chondrocytes.

## 5. Conclusions

In conclusion, the present study showed that the antioxidant efficacy of EESS in chondrocytes is at least involved in the Nrf2/HO-1 signaling pathway-dependent mechanism. Although studies on mitochondrial damage-associated energy metabolism and Nrf2 upstream signal molecules are needed, the results of this study suggest that *S. serratifolium* extract has potential for the prevention and treatment of various diseases caused by oxidative stress. Further studies on the identification of active compounds of *S. serratifolium* and their mechanism of action should be made.

## Abbreviations

ARE	Antioxidant response element
DAPI	4′,6-diamidino-2-phenylindole
DCF-DA	5,6-carboxy-2′,7′-dichlorofluorescin diacetate
DMEM	Dulbecco’s modified Eagle’s medium
DMSO	Dimethylsulfoxide
ECL	Enhanced chemiluminescence
EDTA	Ethylenediaminetetraacetic acid
EESS	Ethanol extract of *S. serratifolium*
EtBr	Ethidium bromide
FBS	Fetal bovine serum
FITC	Fluorescein isothiocyanate
H_2_O_2_	Hydrogen peroxide
HO-1	Heme oxygenase-1
JC-1	5,5′,6,6′-tetrachloro-1,1′,3,3′-tetraethyl-imidacarbocyanine iodide
Keap1	Kelch-like ECH-associated protein-1
MMP	Mitochondrial membrane potential
MTT	3-(4,5-dimethylthiazol-2-yl)-2,5-diphenyltetrazolium bromide
NaCl	Sodium chloride
NaOH	Sodium hydroxide
Nrf2	Nuclear factor-erythroid 2 related factor 2
PARP	Poly (ADP-ribose) polymerase
PBS	Phosphate buffered saline
PI	Propidium iodide
ROS	Reactive oxygen species
SDS	Sodium dodecyl sulfate
ZnPP	Zinc protoporphyrin

## References

[1] Lepetsos P., Papavassiliou A.G. (2016). ROS/oxidative stress signaling in osteoarthritis. Biochim. Biophys. Acta.

[2] Li D., Xie G., Wang W. (2012). Reactive oxygen species: The 2-edged sword of osteoarthritis. Am. J. Med. Sci..

[3] Hwang H.S., Kim H.A. (2015). Chondrocyte apoptosis in the pathogenesis of osteoarthritis. Int. J. Mol. Sci..

[4] Johnson E.O., Charchandi A., Babis G.C., Soucacos P.N. (2008). Apoptosis in osteoarthritis: Morphology, mechanisms, and potential means for therapeutic intervention. J. Surg. Orthop. Adv..

[5] Portal-Núñez S., Esbrit P., Alcaraz M.J., Largo R. (2016). Oxidative stress, autophagy, epigenetic changes and regulation by miRNAs as potential therapeutic targets in osteoarthritis. Biochem. Pharmacol..

[6] Wu L., Liu H., Li L., Liu H., Cheng Q., Li H., Huang H. (2014). Mitochondrial pathology in osteoarthritic chondrocytes. Curr. Drug Targets.

[7] Collins J.A., Diekman B.O., Loeser R.F. (2018). Targeting aging for disease modification in osteoarthritis. Curr. Opin. Rheumatol..

[8] Marchev A.S., Dimitrova P.A., Burns A.J., Kostov R.V., Dinkova-Kostova A.T., Georgiev M.I. (2017). Oxidative stress and chronic inflammation in osteoarthritis: Can NRF2 counteract these partners in crime?. Ann. N. Y. Acad. Sci..

[9] Kang K.A., Hyun J.W. (2017). Oxidative stress, Nrf2, and epigenetic modification contribute to anticancer drug resistance. Toxicol. Res..

[10] Vanella L., Barbagallo I., Tibullo D., Forte S., Zappalà A., Li Volti G. (2016). The non-canonical functions of the heme oxygenases. Oncotarget.

[11] Surh Y.J., Kundu J.K., Li M.H., Na H.K., Cha Y.N. (2009). Role of Nrf2-mediated heme oxygenase-1 upregulation in adaptive survival response to nitrosative stress. Arch. Pharm. Res..

[12] Ndisang J.F. (2017). Synergistic interaction between heme oxygenase (HO) and nuclear-factor E2-related factor-2 (Nrf2) against oxidative stress in cardiovascular related diseases. Curr. Pharm. Des..

[13] Lee D.H., Park J.S., Lee Y.S., Sung S.H., Lee Y.H., Bae S.H. (2017). The hypertension drug, verapamil, activates Nrf2 by promoting p62-dependent autophagic Keap1 degradation and prevents acetaminophen-induced cytotoxicity. BMB Rep..

[14] Loboda A., Damulewicz M., Pyza E., Jozkowicz A., Dulak J. (2016). Role of Nrf2/HO-1 system in development, oxidative stress response and diseases: An evolutionarily conserved mechanism. Cell. Mol. Life Sci..

[15] Arulselvan P., Fard M.T., Tan W.S., Gothai S., Fakurazi S., Norhaizan M.E., Kumar S.S. (2016). Role of antioxidants and natural products in inflammation. Oxid. Med. Cell. Longev..

[16] Vadalà M., Palmieri B. (2015). From algae to “functional foods”. Clin. Ther..

[17] Ali M.Y., Kim D.H., Seong S.H., Kim H.R., Jung H.A., Choi J.S. (2017). α-glucosidase and protein tyrosine phosphatase 1B inhibitory activity of plastoquinones from marine brown alga *Sargassum serratifolium*. Mar. Drugs.

[18] Oh S.J., Joung E.J., Kwon M.S., Lee B., Utsuki T., Oh C.W., Kim H.R. (2016). Anti-inflammatory effect of ethanolic extract of *Sargassum serratifolium* in lipopolysaccharide-stimulated BV2 microglial cells. J. Med. Food.

[19] Kang C.W., Park M.S., Kim N.H., Lee J.H., Oh C.W., Kim H.R., Kim G.D. (2015). Hexane extract from *Sargassum serratifolium* inhibits the cell proliferation and metastatic ability of human glioblastoma U87MG cells. Oncol. Rep..

[20] Gwon W.G., Lee B., Joung E.J., Choi M.W., Yoon N., Shin T., Oh C.W., Kim H.R. (2015). Sargaquinoic acid inhibits TNF-α-induced NF-κB signaling, thereby contributing to decreased monocyte adhesion to human umbilical vein endothelial cells (HUVECs). J. Agric. Food Chem..

[21] Collins A.R., El Yamani N., Lorenzo Y., Shaposhnikov S., Brunborg G., Azqueta A. (2014). Controlling variation in the comet assay. Front. Genet..

[22] Rogakou E.P., Pilch D.R., Orr A.H., Ivanova V.S., Bonner W.M. (1998). DNA double-stranded breaks induce histone H2AX phosphorylation on serine 139. J. Biol. Chem..

[23] Chin K.Y., Pang K.L. (2017). Therapeutic effects of olive and its derivatives on osteoarthritis: From bench to bedside. Nutrients.

[24] Mammucari C., Rizzuto R. (2010). Signaling pathways in mitochondrial dysfunction and aging. Mech. Ageing Dev..

[25] D’Autréaux B., Toledano M.B. (2007). ROS as signalling molecules: Mechanisms that generate specificity in ROS homeostasis. Nat. Rev. Mol. Cell Biol..

[26] Finkel T., Holbrook N.J. (2000). Oxidants, oxidative stress and the biology of ageing. Nature.

[27] Valero T. (2014). Mitochondrial biogenesis: Pharmacological approaches. Curr. Pharm. Des..

[28] Rigoulet M., Yoboue E.D., Devin A. (2011). Mitochondrial ROS generation and its regulation: Mechanisms involved in H_2_O_2_ signaling. Antioxid. Redox Signal..

[29] Waterhouse N.J., Goldstein J.C., von Ahsen O., Schuler M., Newmeyer D.D., Green D.R. (2001). Cytochrome *c* maintains mitochondrial transmembrane potential and ATP generation after outer mitochondrial membrane permeabilization during the apoptotic process. J. Cell Biol..

[30] Wu C.C., Bratton S.B. (2013). Regulation of the intrinsic apoptosis pathway by reactive oxygen species. Antioxid. Redox Signal..

[31] Kadenbach B., Arnold S., Lee I., Hüttemann M. (2004). The possible role of cytochrome *c* oxidase in stress-induced apoptosis and degenerative diseases. Biochim. Biophys. Acta.

[32] Lindsay J., Esposti M.D., Gilmore A.P. (2011). Bcl-2 proteins and mitochondria-specificity in membrane targeting for death. Biochim. Biophys. Acta.

[33] Kiraz Y., Adan A., Kartal Yandim M., Baran Y. (2016). Major apoptotic mechanisms and genes involved in apoptosis. Tumour Biol..

[34] Ha A.W., Kim W.K. (2017). Antioxidant mechanism of black garlic extract involving nuclear factor erythroid 2-like factor 2 pathway. Nutr. Res. Pract..

